# Multiphysics Coupling Simulation and Parameter Study of Planar Solid Oxide Fuel Cell

**DOI:** 10.3389/fchem.2020.609338

**Published:** 2021-01-22

**Authors:** Zheng Dang, Xin Shen, Jinyan Ma, Zhaoyi Jiang, Guang Xi

**Affiliations:** ^1^ Department of Fluid Machinery and Engineering, School of Energy and Power Engineering, Xi’an Jiaotong University, Xi’an, China; ^2^ Department of Civil Engineering, School of Human Settlement and Civil Engineering, Xi’an Jiaotong University, Xi’an, China

**Keywords:** solid oxide fuel cells, multiphysics coupling, physical fields distribution, parameter study, work performance

## Abstract

In this paper, a numerical model of gas flow, heat transfer, mass transfer and electrochemical reaction multi-physics field coupling of a planar SOFC is established and solved. According to the calculation results, the distribution of velocity, temperature and concentration inside the SOFC cell is analyzed. The influence of cathode inlet flow rate, porosity, rib width and other parameters on the performance of SOFC is also discussed. The results show that within a certain range, increasing the cathode inlet flow rate can significantly increase the average current density of the cell. Increasing the porosity of the electrode can improve the gas diffusion of the porous electrode, thereby increasing the rate of the electrochemical reaction. Increasing the width of the ribs will result in a significant decrease in cell performance. Therefore, the rib width should be reduced as much as possible within the allowable range to optimize the working performance of the cell.

## Introduction

As a new energy technology, anode supported planar solid oxide fuel cell (SOFC) has attracted much attention in recent years (Kong et al., 2016; Wang et al., 2018a; Wang et al., 2018b). Compared with other types of fuel cells, SOFC has the advantages of diversified fuel selection, high power generation efficiency, and no need for precious metal catalysis (Zheng et al., 2015; Presto et al., 2018; Zhang et al., 2018; Futamura et al., 2019; Hagen et al., 2019; Sahli et al., 2019). In order to further improve the performance of SOFC, a detailed understanding of its specific working process is required (Kong et al., 2020). The operation of SOFC involves a variety of physical processes such as heat transfer, material transport, fluid flow and chemical and electrochemical reactions. These physical processes are highly coupled and interact with each other, and their internal velocity field, temperature field and electric field are extremely complex. In general, experimental methods for SOFC are costly and time-consuming because of its multiscale feature of the porous electrodes. In contrast, the numerical simulation based upon computational fluid dynamics (CFD) method is much cheaper and more efficient, which can easily change various parameters of SOFC and analyze their impact on SOFC performance. Therefore, numerical simulation method for SOFC is widely used.

Ferguson et al. (1996) established three-dimensional mathematical models of SOFC of flat and tubular structures, and studied and analyzed the effects of three different flow modes, namely, codirectional flow, reverse flow and cross flow, on electric field, temperature field and concentration field. Iwata et al. (2000) designed a quasi-two (co-flow and counter-flow) and quasi-three (cross-flow) dimensional simulation program considering mass, charge and heat balance, and obtained the temperature and current density distribution along the flow direction and perpendicular to the electrolyte membrane. Jeon et al. (2006) established a two-dimensional reaction interval model containing a complete single channel, and optimized the width ratio of the flow channel and the interconnector through the research results. Stygar et al. (2012) established a 3-dimensional corrugated SOFC model, and studied the influence of the unit geometry on the temperature distribution and heat transfer rate in the interconnector. The influence of co-current and counter-current on temperature distribution is considered in the simulation. The results show that the temperature gradient is relatively low and the temperature distribution is relatively uniform during counter-flow, which has a significant impact on the electrical conductivity, corrosion performance and durability of the connector. Yuan et al. (Andersson et al., 2012) comprehensively considered the physical processes such as heat transfer, mass transfer, fluid flow, charge transport, internal reforming reaction of fuel and electrochemical reaction, and coupled multiple physical fields to build a numerical model of SOFC single channel at medium temperature. The effects of ion and electron transport resistance in the electrode as well as operating temperature and excessive airflow on the cell performance were studied. Zhang et al. (2015) established an anode-supported three-dimensional SOFC single channel model based on computational fluid dynamics (CFD), and compared the calculated results with the experimental results, which were well verified. The distribution of temperature, reactant velocity, current density and reactant concentration in different flow modes is explained in detail by comparing three different flow modes (co-, counter- and cross-flow). It is found that the output power of the current and counter-flow mode is much higher than that of the cross-flow mode when other operating parameters are kept constant. Dong (2019) established a complete SOFC multi-physics coupling model based on the open-source fuel cell code, and used the model to predict the performance of the cell, and discussed the transmission phenomenon and electrochemical characteristics inside the cell. Zeng et al. (2018) developed a three-dimensional model of the solid oxide fuel together with the optimized interconnection design. The results show that the current density and thermal stress are related to the shape of the interconnection tip and the depth of the cathode. Compared with rectangular and quadrilateral tip interconnects, triangular tip interconnects have the best electrochemical performance. With the increase of the tip depth, the current density will increase accordingly, and only the current density of the trapezoidal ribs decreases with the increase of the tip depth. Wang et al. (2018a), Wang et al. (2018b) established a three-dimensional model of a planar anode supported solid oxide fuel cell, and the influence of working pressure on the cell performance was studied. The results indicate that increasing the operating pressure can improve the cell performance by increasing the open circuit voltage and reducing the activation overpotential, and enhance the electrochemical reaction near the electrolyte. Lee et al. (2017) presented a three-dimensional model of a reversible solid oxide fuel cell, and the effects of different geometric structures and operating parameters (electrode support layer thickness; interconnector rib size; fuel gas composition) on current-potential characteristics and round-trip efficiency were studied. The results show that due to the uneven distribution of the reactants, the size of the ribs has a significant impact on the cell performance, especially when the support layer is thin. Li et al. (2018) proposed a three-dimensional model that couples mass transfer, electron transport, and electrochemical reactions based on a solid oxide fuel cell with porous electrodes and gas channels, the results show that the current density distribution is significantly related to the gas composition distribution. Meanwhile, the simulation results also indicate that, the ridgeline of the interconnection line will limit the mass transfer inside the cell and negatively affect the electrochemical reaction.

However, the current research rarely involves the influence of the rib width parameter of the cell on the cell performance. Only the research by Li et al. (2018) considered the rib width parameter without considering the effect of temperature field in the simulation. The present work discussed the influence of rib width with models coupled with temperature field. In this research, a three-dimensional multi-physics model of planar SOFC is established to study the physical fields distribution in the cell. And the effect of some operation parameters such as cathode inlet flow rate, inlet temperature on the cell is discussed based on the model. Meanwhile, the cell performance under different structural parameters (porosity and rib width) was studied, the rib width is an important cell structural parameter.

## Model Geometry

**FIGURE 1:**
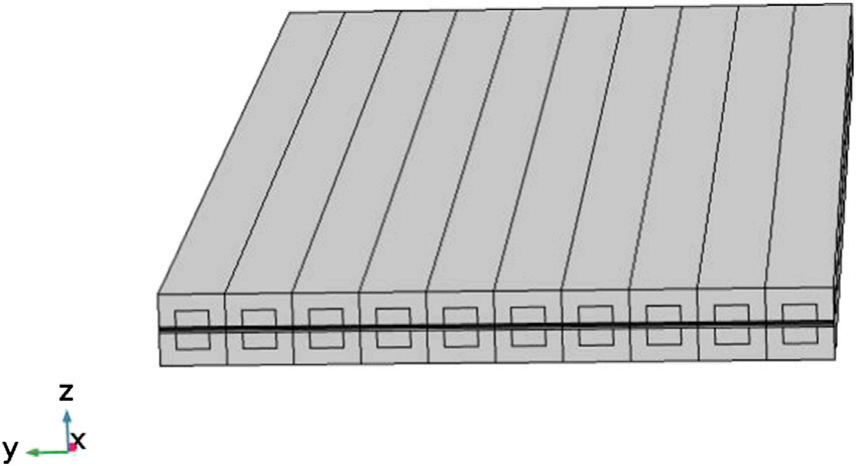
Schematic figure of the SOFC model.

A three-dimensional planar SOFC cell is shown in Figure 1. It contains 10 fuel and air channels. The flow direction of air and fuel in this cell are counter-flow.

This model was established for result verification and parameter study and its geometry parameters are selected based on the work by Khazaee et al. (Iman, 2017), as defined in Table 1.

**TABLE 1:** SOFC cell geometry (Iman, 2017).

Geometry parameter	Value/mm
Gas channel width	2
Gas channel height	1
Interconnector height	2
Width of interconnect	2
Gas channel length	100
Anode thickness	0.15
Electrolyte thickness	0.1
Cathode thickness	0.1

## Governing Equations

### Electrochemical Model

For hydrogen fuel cells, hydrogen and oxygen are fed into the anode and cathode channels of the cell respectively. When hydrogen and oxygen pass through the electrode surface, they diffuse to the electrode/electrolyte interface through the porous electrode structure, and the oxygen is at the cathode and electrolyte interface. Obtain electrons to form oxygen ions. The oxygen ions are transferred to the anode/electrolyte interface through the solid electrolyte layer, and chemically react with the hydrogen on it, and chemically react with the hydrogen on it to generate water vapor, while the oxygen ions lose electrons; The external circuit conducts from the anode to the cathode, forming a closed loop and generating current at the same time. The electrochemical reactions occurring at the anode and cathode of the cell are as follows:

$$\begin{array}{l} \text{Anode:}\\ {\text{H}}_{2}+{\text{O}}^{2-}\to {\text{H}}_{2}\text{O}+2{\text{e}}^{-} \end{array}$$ (1)

$$\begin{array}{l} \text{Cathode}:\\ \frac{1}{2}{\text{O}}_{2}+2{\text{e}}^{-}\to {\text{O}}^{2-} \end{array}$$ (2)

The electrical energy output by the solid oxide fuel cell is converted from the chemical energy of the fuel. It can be known from the thermodynamic formula that when the temperature and pressure remain unchanged, the maximum output electrical work of the SOFC is equal to the decrease in Gibbs free energy. Thus, the open circuit voltage (EOCV) of SOFC can be obtained, that is, the Nernst potential is as the following:

$$E_{Nernst}=-\frac{\Delta G\left(T,P\right)}{2F}=-\frac{\Delta G\left(T,P_{0}\right)}{2F}\ln\left(\frac{P_{H_{2}}P_{O_{2}}^{0.5}}{P_{H_{2}O}}\right)=E_{Nernst}^{0}+\frac{RT}{2F}\ln\left(\frac{P_{H_{2}}P_{O_{2}}^{0.5}}{P_{H_{2}O}}\right)$$ (3)

where $E_{Nernst}$ denotes Nernst potential(V), ΔG(T,P) denotes Gibbs free energy under the condition of temperature *T* and pressure *P*, $P_{H_{2}}$ denotes hydrogen partial pressure at the three-phase interface (Pa), $P_{O_{2}}$ denotes oxygen partial pressure at the three-phase interface, $P_{H_{2}O}$ denotes the partial pressure of water vapor at the three-phase interface.

In the actual working process of SOFC, there are some resistances that make the actual cell output voltage less than the ideal voltage $E_{Nernst}$. This voltage loss phenomenon is called polarization. The main polarization losses are divided into three types, namely activation polarization, concentration polarization and ohmic polarization, defined as follows:

$$\eta_{act}=\frac{2RT}{nF}\sin h^{-1}\left(\frac{i}{2i_{0}}\right)$$ (4)

$$\eta_{conc,a}=\frac{RT}{2F}\ln\left(\frac{P_{H_{2}}^{0}P_{H_{2}O}^{tpb}}{P_{H_{2}}^{tpb}P_{H_{2}O}^{0}}\right)$$ (5)

$$\eta_{conc,c}=\frac{RT}{4F}\ln\left(\frac{P_{O_{2}}^{0}}{P_{O_{2}}^{tpb}}\right)$$ (6)

$$\eta_{ohm}=i\cdot R$$ (7)

$$i=i_{0}\{\exp\left(\frac{\alpha nF}{RT}\eta_{act}\right)-\exp\left(\frac{-\left(1-\alpha \right)nF}{RT}\eta_{act}\right)\}$$ (8)

where $\eta_{act}$ denotes activation polarization, $\eta_{conc,a}$ denotes concentration polarization of anode, $\eta_{conc,c}$ denotes concentration polarization of cathode, $\eta_{ohm}$ denotes ohmic polarization, and i denotes the local current density (A·m^−2^), $i_{0}$ denotes the reference exchange current density (A·m^−2^). $i_{0}$ can be expressed by Equation (8), where $k_{e}$ is the pre-exponential factor, $E_{a}$ is the activation energy.

$$i_{0}=\frac{RT}{nF}\cdot k_{e}\cdot \exp\left(\frac{-E_{a}}{RT}\right)$$ (9)

The actual working potential of SOFC is defined as follows:

$$V_{op}=E_{Nernst}-\eta_{act}-\eta_{conc}-\eta_{ohm}$$ (10)

where $V_{op}$ denotes the actual working potential.

During the operation of SOFC, the charge is transferred in the electrodes, electrolytes and interconnectors in the form of electronic current and ionic current. The transport equations of electron current and ion current are expressed as follows:

$$\nabla \cdot i_{k}=\nabla \left(-\sigma_{k}^{eff}\nabla \varphi_{k}\right)=S_{current}$$ (11)

$$S_{current}=A_{v}\cdot i$$ (12)

where $S_{current}$ denotes a general source term, *k* denotes an index that is *el* for the electrode or *io* for the electrolyte, $A_{v}$ denotes surface area to volume ratio/m^2^·m^−3^, $\varphi_{k}$ the potential (SI unit: V) and $\sigma_{k}^{eff}$ denotes the effective conductivity (SI unit: S/m) which can be calculated as:

$$\sigma_{s,a}^{eff}=\frac{9.5\times 10^{7}}{T}\cdot \exp\left(\frac{-1150}{T}\right)\cdot \frac{V_{Ni,a}}{\tau_{Ni,a}}$$ (13)

$$\sigma_{s,c}^{eff}=\frac{4.2\times 10^{7}}{T}\cdot \exp\left(\frac{-1200}{T}\right)\cdot \frac{V_{LSM,c}}{\tau_{LSM,c}}$$ (14)

$$\sigma_{l,el}^{eff}=3.34\times 10^{4}\cdot \exp\left(\frac{-10300}{T}\right)\cdot \frac{V_{YSZ,el}}{\tau_{YSZ,el}}$$ (15)

where *V* is the volume fraction for the specific materials in the electrodes and t is the structure-dependent tortuosity factors. Table 2 shows the input parameters of the electrochemical model.

**TABLE 2:** Input parameters of electrochemical model (Hajimolana et al., 2011).

Input parameters	Anode	Cathode
*k*_*e*_	6.54 × 10^11^	2.35 × 10^11^
*E*_a_/10^3^ × J⋅mol^−1^	140	137
*n*	2	4
$A_{v}$	5 × 10^5^	5 × 10^5^
$V_{YSZ}$	0.42	0.42
$V_{Ni}/V_{LSM}$	0.28	0.28
ε	0.4	0.4
τ	10	10

### Mass Transport

The material transport process is mainly composed of diffusion and convection. Factors such as concentration gradients, temperature gradients, pressure gradients, etc. will drive the transfer of gas components. At present, there are mainly three models used to describe the mass transfer process, namely the Fick model, the Maxwell–Stefen model and the Dusty gas model. The mass transfer model used in this paper is the Maxwell–Stefen model, which is expressed as follows:

$$\nabla \left(-\rho \cdot \omega_{i}\sum^{\text{​}}D_{eff,ij}\cdot \nabla x_{j}+\left(x_{j}-\omega_{j}\right)\frac{\nabla p}{p}\cdot \vec{u}-D_{i}^{T}\cdot \frac{\nabla T}{T}\right)+\rho \cdot \vec{u}\cdot \nabla \omega_{j}=S_{i}$$ (16)

$$S_{i}=-\frac{i}{2F}M_{i}$$ (17)

where $\omega_{i}$ denotes the mass fraction of material *i*, $D_{i}^{T}$ the thermal diffusion coefficient (assumed to be zero at this work), $S_{i}$ the source term, $M_{i}$ the molar mass of material *i*, and $D_{eff,ij}$ is the effective diffusion coefficients, which can be calculated as follows:

$$D_{eff,ij}=\varepsilon^{\tau }\cdot D_{ij}$$ (18)

where $D_{ij}$ denotes the binary diffusion coefficient, and can be expressed by Eq. (16):

$$D_{ij}=\frac{k_{d}\cdot T^{1.75}\cdot {\left(\frac{1}{M_{i}}+\frac{1}{M_{j}}\right)}^{0.5}}{p\cdot {\left(v_{i}^{\frac{1}{3}}+v_{j}^{\frac{1}{3}}\right)}^{2}}$$ (19)

### Momentum Transport

The Navier–Stokes equation is usually used to describe the flow process in the flow channel in SOFC. The combination of the N–S equation and the continuity equation can be expressed as:

$$\left(\rho \vec{u}\cdot \nabla \right)\vec{u}=-\nabla p+\nabla \cdot \left[\mu \left(\nabla \vec{u}+{\left(\nabla \vec{u}\right)}^{T}\right)-\frac{2}{3}\mu \left(\nabla \cdot \vec{u}\right)I\right]\nabla \left(\rho \vec{u}\right)$$ (20)

$$\nabla \cdot \left(\rho \vec{u}\right)=0$$ (21)

where ρ denotes density (kg m^−3^), μ the viscosity coefficient of fluid (kg m^−1^ s^−1^).

The momentum transfer in porous media can be described by the Darcy–Brinkman equation. The combination of Brinkman equation and continuity equation can be expressed as:

$$\frac{\mu }{\kappa }\vec{u}=-\nabla p+\nabla \cdot \frac{1}{\varepsilon }\left[\mu \left(\nabla \vec{u}+{\left(\nabla \vec{u}\right)}^{T}\right)-\frac{2}{3}\mu \left(\nabla \cdot \vec{u}\right)I\right]$$ (22)

$$\nabla \cdot \left(\rho \vec{u}\right)=S_{i}$$ (23)

where κ is the permeability of the porous medium (m^2^), $S_{i}$ the source term.

### Heat Transport

To accurately simulate the actual working process of the SOFC, thereby optimizing cell performance and improving thermal stability, the internal energy transfer process of the SOFC must be considered.

For the solid area (electrolyte and connecting body), only heat conduction needs to be considered, the thermal energy equation can be expressed as:

∇⋅(−k∇T)=S (24)

For the flow channel, heat conduction and convection heat transfer need to be considered at the same time, the thermal energy equation can be expressed as:

$$\nabla \cdot \left(-k\nabla T+C_{f}C_{p}T\vec{u}\right)=S$$ (25)

For the porous electrode area, the local thermal equilibrium assumption is generally adopted, that is, the temperature of the porous medium material in the local area is equal to the temperature of the fluid, so the energy governing equation is:

$$\rho_{g}\cdot \varepsilon \cdot C_{p,g}\cdot \vec{u}\cdot \nabla T=\nabla \cdot \left(k_{eff}\nabla T\right)+S$$ (26)

where $C_{f}$ is the molar concentration of fluid, $C_{p,g}$ the specific heat of gas mixture, $\rho_{g}$ the density of gas mixture, *S* the source term, and $k_{eff}$ effective thermal conductivity.

The source term *S* for the thermal energy equation due to the electrochemical reactions can be expressed as:

$$S=\left|i\right|\cdot \left(\frac{T\cdot \left|\Delta S\right|}{nF}+\left|\eta_{act}\right|+\eta_{conc}\right)+\sum^{\text{​}}\frac{i^{2}}{\sigma }$$ (27)

### Input Parameters and Boundary Conditions

The operating conditions and input parameters for the developed model are listed in Table 3. The potential at the anode current collector is set to zero and at of the cathode current collector is as the cell operating voltage. All other external boundaries and interfaces are insulated.

**TABLE 3:** Input parameters.

Parameters	Symbol	Value
Anode thermal conductivity/W m^−1^ K^−1^	$k_{s,a}$	11
Cathode thermal conductivity/W m^−1^ K^−1^	$k_{s,c}$	6
Electrolyte thermal conductivity/W m^−1^ K^−1^	$k_{s,el}$	2.7
Interconnect thermal conductivity/W m^−1^ K^−1^	$k_{\mathrm{int}}$	20
Anode specific heat/J kg^−1^ K^−1^	$C_{p,a}$	450
Cathode specific heat/J kg^−1^ K^−1^	$C_{p,c}$	430
Electrolyte specific heat/J kg^−1^ K^−1^	$C_{p,el}$	470
Interconnect specific heat/J kg^−1^ K^−1^	$C_{p,\mathrm{int}}$	550
Anode density/kg m^−3^	$\rho_{a}$	3.31 × 10^3^
Cathode density/kg m^−3^	$\rho_{c}$	3.03 × 10^3^
Electrolyte density/kg m^−3^	$\rho_{el}$	5.16 × 10^3^
Interconnect density/kg m^−3^	$\rho_{\mathrm{int}}$	3.03 × 10^3^
Operating pressure/Pa	$p_{0}$	1.01 × 10^5^
Operating temperature/K	$T_{operate}$	1,073
Inlet air velocity/m·s^−1^	$u_{a}$	3
Inlet fuel velocity/m s^−1^	$u_{c}$	0.4
Hydrogen inlet mass fraction	$\omega_{H_{2}}$	0.4
Oxygen inlet mass fraction	$\omega_{O_{2}}$	0.15
Inlet temperature/K	$T_{in}$	1,073

## Result and Discussion

**FIGURE 2:**
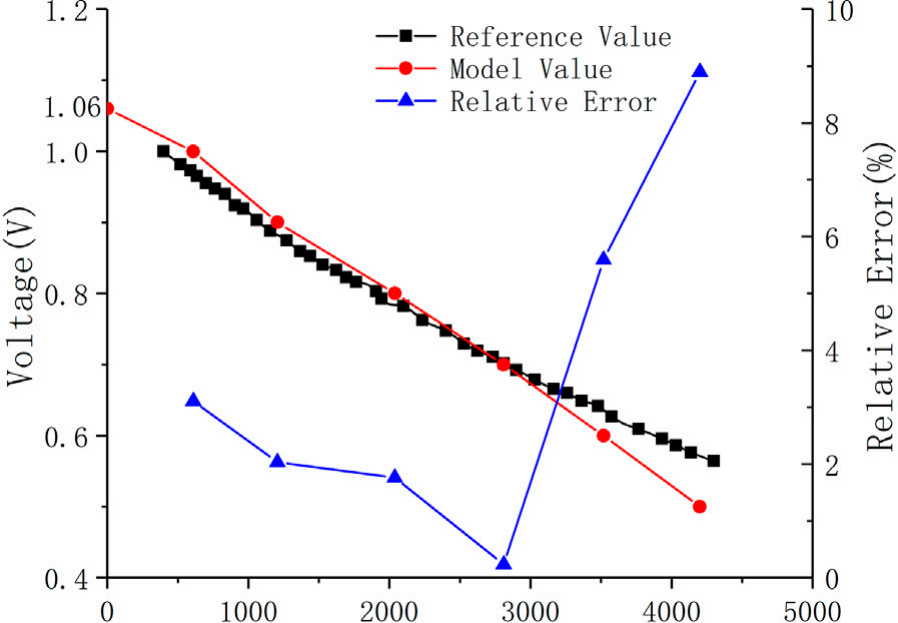
Comparison of the model-predicted V–I curve with experimental data at the same operating condition.

In order to prove the correctness of the model built in this paper, the simulated calculation results are compared with the experimental data provided by Yakabe et al. (2001). in the literature. The comparison results of the volt-ampere characteristic curves are shown in Figure 2. The results show that the calculation results of the planar SOFC model established in this paper are in good agreement with the experimental data given in the literature, and the maximum error is less than 10%. Therefore, the model can be used to study the performance of SOFC.

### Physical Fields Distribution

#### Temperature Distribution

**FIGURE 3:**
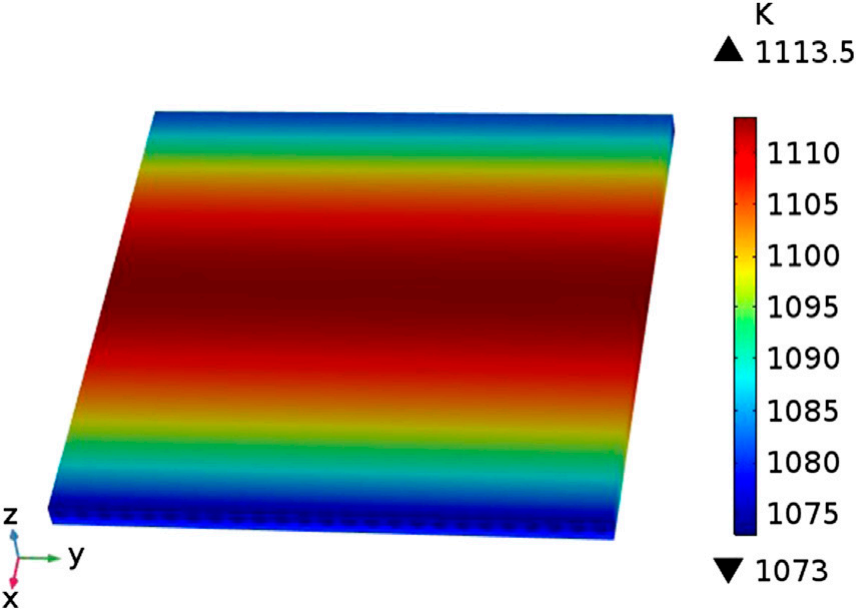
The distribution of the temperature field.

The distribution of the temperature field inside the cell in the case of counter-flow is shown in Figure 3. It can be seen that the cell has a low temperature (1073 K) at both ends and a high temperature (around 1110 K) in the middle. This is because the fuel gas and air in the model are in a reverse flow mode, and the inflow temperature is both 1073 K. With the continuous inflow of fuel gas and air Inside the cell, the heat generated by the electrochemical reaction continues to accumulate, and the cell temperature rises accordingly, reaching a maximum near the middle of the cell. At both ends of the cell, the high-temperature gas flowing out of the cell exchanges heat with the gas flowing into the other side of the cell, and the temperature gradually drops.

#### Velocity Field Distribution

**FIGURE 4:**
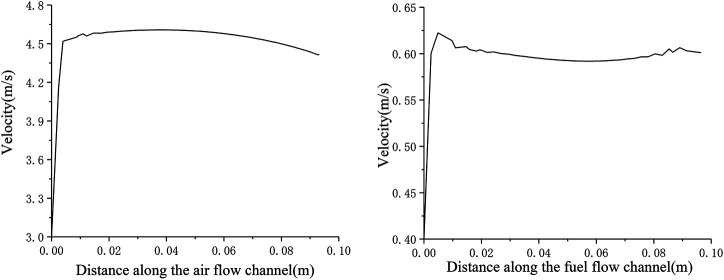
Velocity distribution in flow channel.

It can be seen from Figures 4A, B that the inlet velocity of the anode fuel gas is 0.4 m s^−1^. After entering the cell, the boundary layer is formed first, and the flow velocity rapidly increases to about 0.58 m s^−1^. At a relatively stable level, along the fuel flow direction, the hydrogen flow velocity in the anode flow channel hardly changes; while the cathode air inlet velocity is 3 m s^−1^ at the inlet. After entering the cell, the boundary layer is also formed. The flow velocity rapidly increased to about 4.5 m s^−1^, and then the gas flow velocity in the cathode flow channel decreased continuously along the air flow direction. There is a difference in the velocity field distribution of the cathode and anode flow channels. This is because the anode consumes 1 mol of hydrogen and generates 1 mol of water vapor, while the total molar flow rate of the mixed gas inside the anode remains unchanged, so the velocity is almost unchanged; The continuous consumption of the flow direction causes the total molar flow of the cathode gas to be continuously reduced, and therefore the flow rate of cathode fluid is continuously reduced.

#### Concentration Field Distribution

**FIGURE 5:**
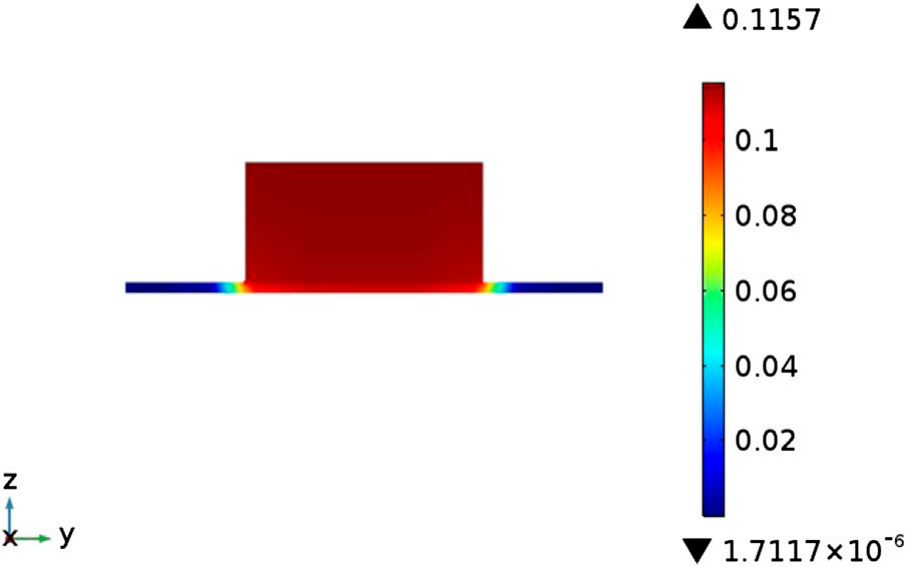
Oxygen concentration distribution in anode flow channel.

The oxygen concentration distribution on the cross section of the cell are shown in Figure 5. It can be seen from Figure 5 that the oxygen concentration in the flow channel has been maintained at a high level (around 0.1). The oxygen concentration inside the porous electrode directly in contact with the flow channel is also relatively high, but the oxygen concentration in the cell ribs (the part of the porous electrode directly in contact with the interconnector) is very low (less than 0.01). There are three main reasons for this phenomenon: First, It is because the oxygen concentration in the flow channel is low (the oxygen inlet mole fraction is 0.14); secondly, the rib area does not directly contact the flow channel, so its oxygen transportation mainly depends on diffusion, and the binary diffusion coefficient of oxygen is lower than other gas in SOFC, which is not conducive to the diffusion of oxygen, and for common anode-supported SOFCs, the thickness of the cathode is generally thin, which will also make the diffusion of oxygen more difficult. The imbalance of the oxygen concentration distribution will lead to the imbalance of the electrochemical reaction, resulting in uneven temperature distribution inside the cell.

### Concentration Field Distribution

**FIGURE 6:**
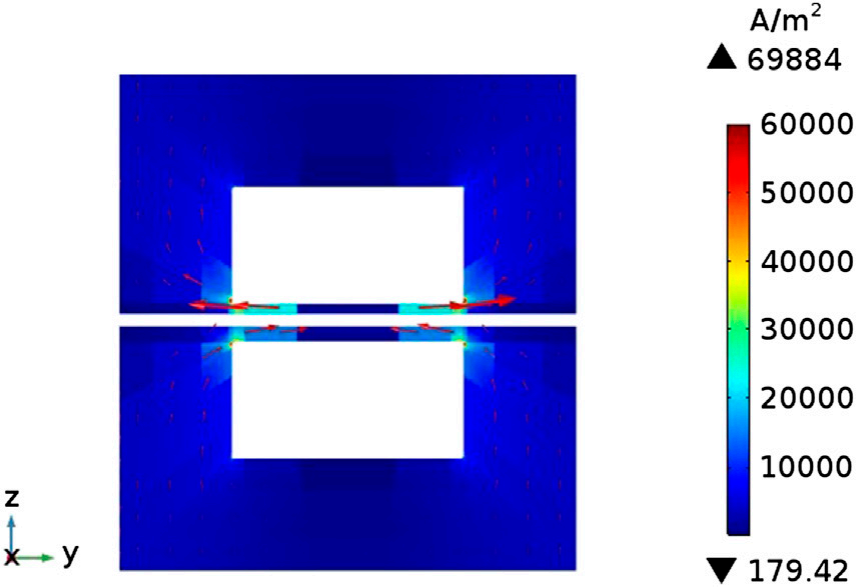
Current density distribution on the cross section of the cell.

Figure 6 shows the current density distribution on the cross section of the flow channel. The arrow direction represents the direction of current flow. It can be known that the current density distribution on the entire cross-section is relatively uniform and the values are relatively small (less than 1 × 10^5^ A m^−2^), but near the contact surface of the flow channel and the interconnector, the current density has a maximum value around 7 × 10^5^ A m^−2^. This is because the electronic current in the electrode can only pass through the interconnector part, and always takes the shortest path, but the flow channel is not conductive, therefore, the electronic current generated by the porous electrode part will be transmitted laterally to both sides of the flow channel, and flows into the interconnector at the position where he flow channel is in contact with interconnector.

### Parameter Study

The change of SOFC's working parameters and structural parameters will affect the distribution of physical fields inside the cell. This part mainly studies the influence of the several parameters on the performance of SOFC. The research results are calculated under the condition of output voltage *V*_*op*_ = 0.7 V.

#### Influence of Cathode Inlet Flow Rate

**FIGURE 7:**
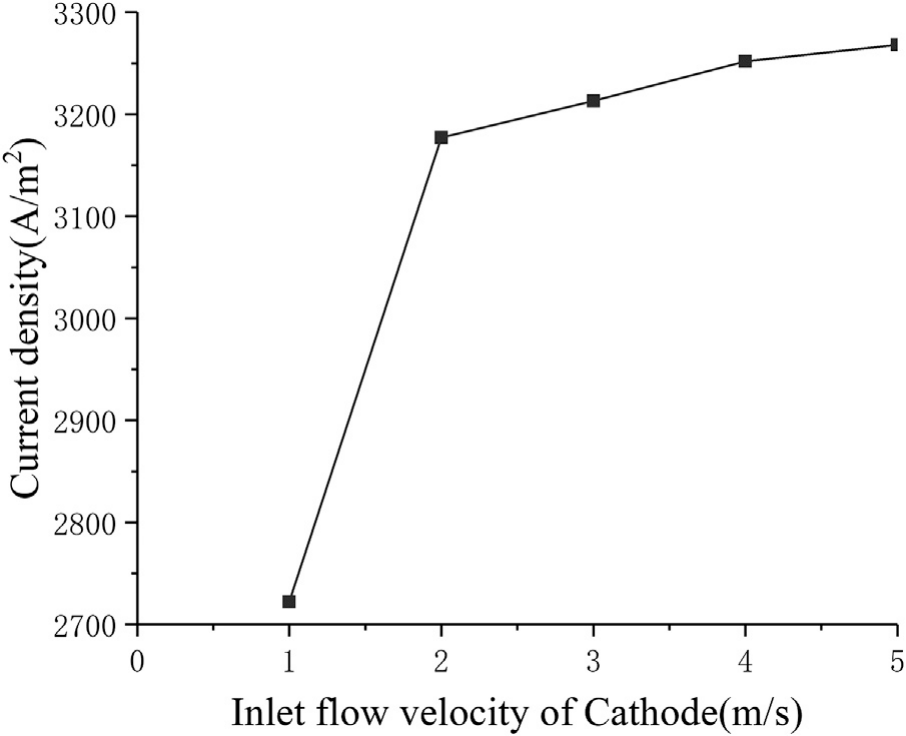
The relationship between current density and cathode inlet flow rate.

In order to study the influence of the cathode inlet flow rate on the performance of SOFC, the multi-physical field distribution of the cathode flow rate from 1 to 5 m s^−1^ was calculated. Figure 7 shows the change in current density when the cathode inlet flow rate changes (the standard operating condition is the cathode inlet flow rate *u*_*c*_ = 3 m s^−1^). It can be seen that when the cathode flow rate increases from 1 to 2 m s^−1^, the current density increases rapidly from around 2700 A m^−2^ to around 3200 A m^−2^. When the cathode inlet flow rate continues to increase, the increase in current density gradually slows down. This is because the initial air inlet flow is too small, so the oxygen in the back of the cell is exhausted. The main reason for limiting the cell performance is insufficient supply of reactants. At this time, increasing the cathode air flow will increase the current density significantly. As the cathode inlet flow rate continues to increase, the air on the cathode side gradually becomes excessive, but the electrochemical reaction in the cell has already obtained enough reactants, so the improvement of cell performance is not obvious, When the inlet flow velocity of Cathode increases from 2 to 5 m s^−1^, the current density only increases by less than 100 A m^−2^.

**FIGURE 8:**
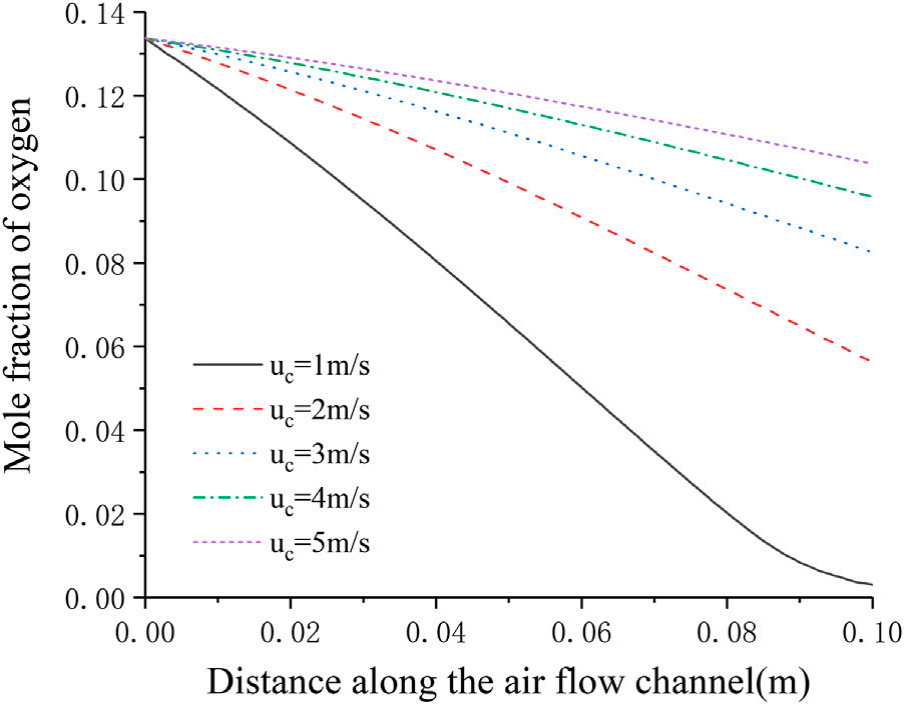
Distribution of oxygen concentration in the cathode flow channel under different cathode inlet flow rates.

Figure 8 shows the distribution of oxygen concentration in the flow channel under different cathode inlet flow rates. It can be seen that the lower the cathode inlet flow rate, the longer the oxygen stays in the cell, and the more oxygen is consumed. When the cathode flow rate is 1 m s^−1^, the downward trend of the oxygen mole fraction at the outlet has a significant slowdown, and is close to 0, indicating that the flow rate of oxygen is not enough under this flow rate condition. On the other hand, with the continuous increase of the cathode inlet flow rate, the consumption of oxygen in the flow channel is getting lower and lower, and Figure 7 shows that the current density is continuously increasing, which shows that after sufficient reactants are provided, increasing the oxygen flow rate mainly improves the diffusion process of oxygen in the porous medium, thereby increasing the current density and improving the cell performance.

#### Influence of Inlet Temperature

**FIGURE 9:**
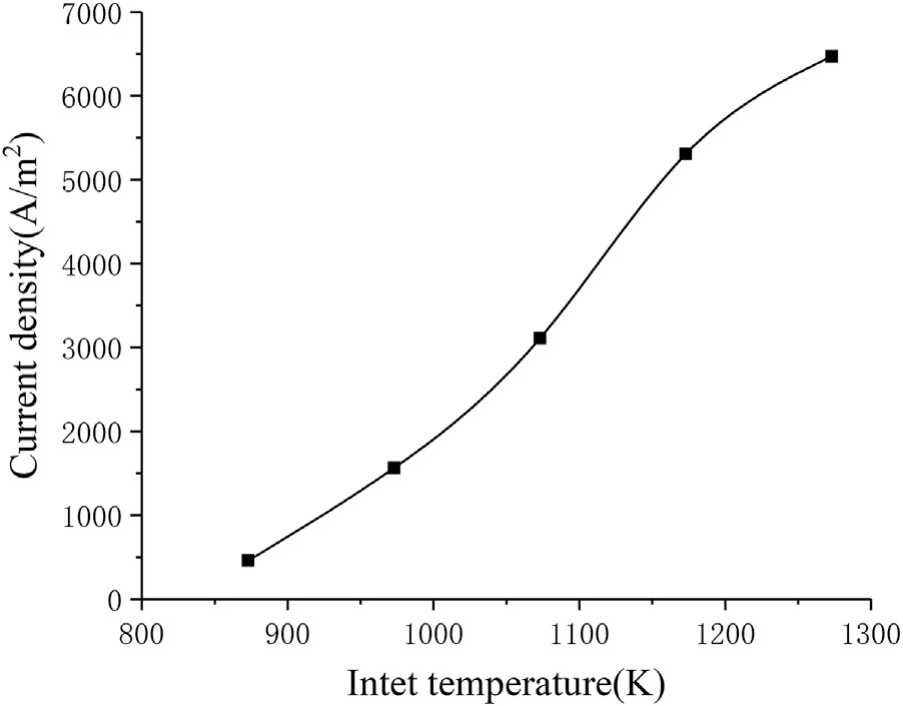
The relationship between inlet temperature and current density.

The electrochemical reaction process in the SOFC working process is greatly affected by temperature. In order to study the influence of the inlet temperature on the performance of the SOFC, In the range of 873–1273 K, every 100 K is selected as a working condition point, a total of five inlet temperature conditions are solved separately. Figure 9 shows the corresponding relationship between inlet temperature and current density. It can be seen that the current density increases significantly as the inlet temperature increases (from around 500 A m^−2^ to around 6500 A m^−2^). This is consistent with the formula given in the electrochemical model, that is, as the temperature rises, the local current density will increase, thereby increasing the average current density of the cell.

#### Influence of Porosity

**FIGURE 10:**
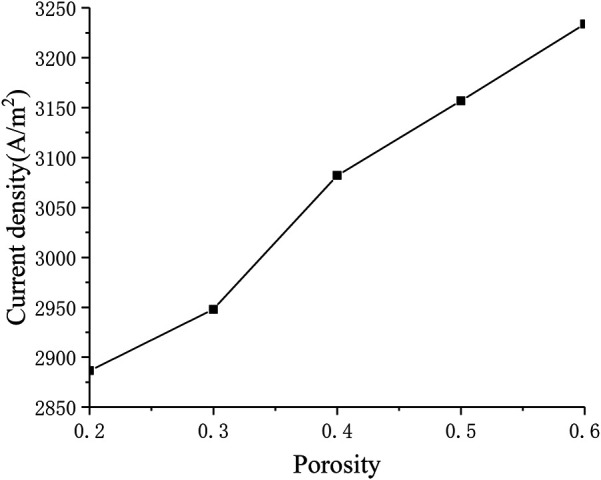
The relationship between porosity and current density.

Porosity is the microstructure parameter of the porous electrode, which reflects the difficulty of gas transfer in the porous medium. Figure 10 describes the corresponding relationship between current density and porosity. It can be seen that as the porosity continues to increase, the current density also increases (from around 2900 A m^−2^ to around 3200 A m^−2^). This is because the porosity improves the diffusion characteristics of the electrode, making the diffusion of oxygen and hydrogen inside the porous electrode easier, thereby improving cell performance.

#### Influence of Rib Width

**FIGURE 11:**
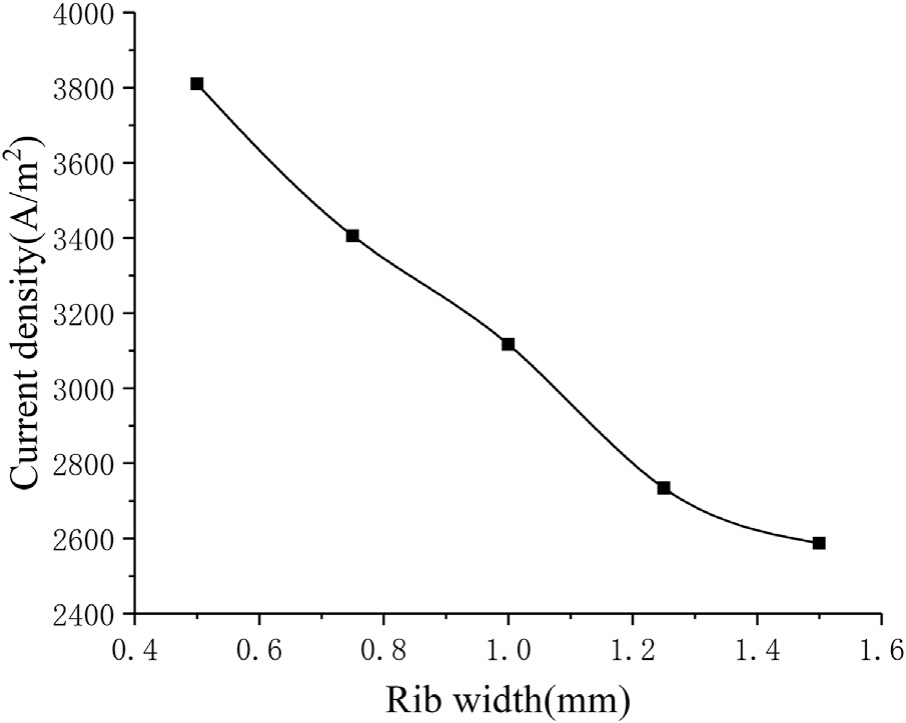
The relationship between rib width and current density.

**FIGURE 12:**
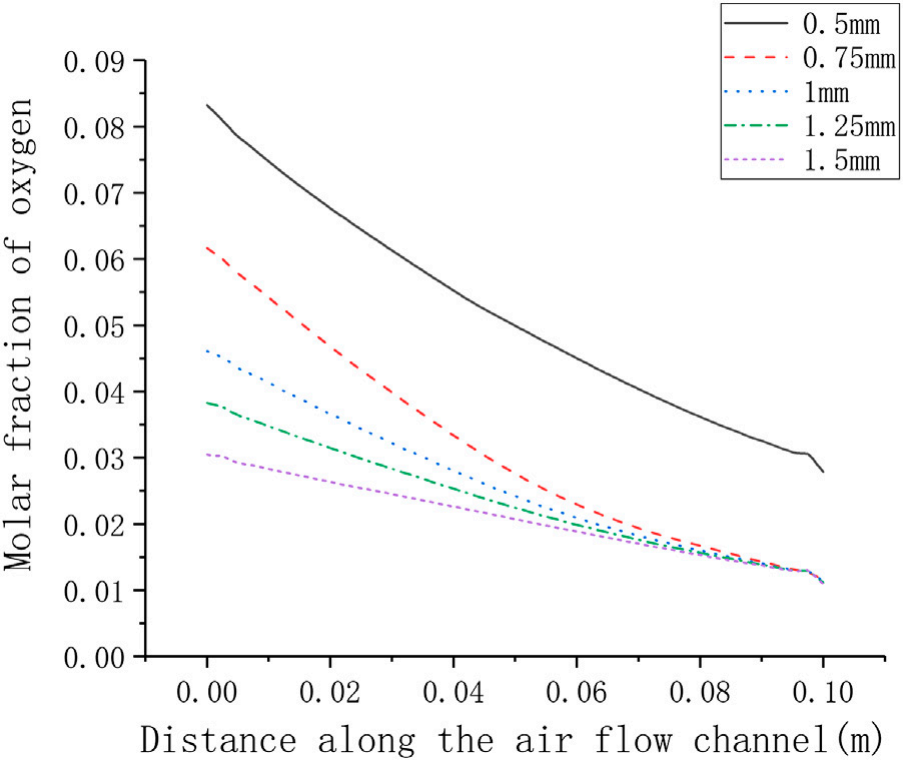
Distribution of oxygen concentration in the rib under different rib width.

The different rib width parameters selected for numerical calculation are 0.5, 0.75, 1.25, 1.5 mm. Figure 11 shows the corresponding relationship between rib width and current density. It can be seen that as the rib width increases, the current density gradually decreases (from around 3800–2600 A m^−2^). In order to understand the gas transport situation of the cell ribs, a straight line is selected as the calculation area on the center plane of the cathode ribs, and the distance between the straight line and one side of the rib is 0.1 times of the whole rib width. For different rib widths, the oxygen concentration distribution on the straight line is shown in Figure 12. Generally, as the rib width increases, the average oxygen concentration in the rib area becomes lower. When the rib width is 0.5 mm, the oxygen mole fraction at the cathode exit is about 3%, and when the rib width is other values, the oxygen mole fraction at the cathode exit is very close, both being about 1%. This is because when the rib width is greater than 0.75 mm, the oxygen concentration near the exit is too low, which is close to the lower limit of concentration preset in the simulation. It can be considered that the oxygen has been completely consumed at this time and has no contribution to the electrochemical reaction. Meanwhile, the larger the rib width, the lower the oxygen concentration at the entrance, that is, the smaller the total contribution of the entire rib area to the cell performance. Therefore, increasing the rib width is equivalent to increasing the area of the adverse reaction zone, on the other hand, increasing the rib width makes the oxygen transmission path in the porous electrode longer, and it is more difficult for oxygen to transport to the middle of the rib. In addition, increasing the rib width will also lengthen the current transmission length in the cathode, which increases the ohmic polarization. Combining the above three factors, increasing the rib width will reduce the performance of the cell. Correspondingly, the performance of the cell can be improved by reducing the rib width. However, the rib width is restricted by the manufacturing process and cost. Considering all factors, reducing the rib width as much as possible can improve the working performance of the cell.

## Conclusion

In this paper, a numerical model of multi-physics coupling of a planar SOFC is established and solved. According to the calculation results, the distribution of various physical parameters inside the SOFC cell is analyzed. The influence of cathode inlet flow rate, porosity, rib width and other parameters on the performance of SOFC is discussed with a fully coupled models. The following conclusions are mainly drawn:

1. Due to the thinner cathode porous electrode and the smaller oxygen binary diffusion coefficient, the oxygen concentration in the electrode area directly in contact with the flow channel is much greater than the oxygen concentration in the electrode area (rib) directly in contact with the interconnector. The concentration varies greatly between the electrode area covered by channel and the electrode area covered by the ribs. This uneven concentration distribution will affect the electrochemical reaction rate; the current density distribution inside the SOFC is not uniform, and its maximum value is located near the interface between the cathode ribs and the flow channel
2. When the cathode inlet flow rate is less than a certain value, the oxygen flowing into the cathode will be exhausted before it reaches the cell outlet. In this range, increasing the cathode inlet flow rate can significantly improve the working performance of the cell. When the cathode inlet flow rate is greater than the critical value, the inlet air can meet the needs of the electrochemical reaction, and it is difficult to increase the inlet flow rate to significantly improve the cell performance.
3. Increasing the porosity can improve the gas diffusion inside the porous electrode and reduce the concentration polarization, thereby increasing the output voltage of SOFC; Increasing the rib width will extend the gas diffusion path and the electron transmission path in the porous electrode. In order to improve the working performance of the cell, the rib width should be reduced as much as possible within the range allowed by objective conditions such as the manufacturing process.

## Data Availability Statement

The original contributions presented in the study are included in the article/Supplementary Material, further inquiries can be directed to the corresponding author.

## Author Contribution

ZD responsible for the overall organization, conception and writing of the paper. XS responsible for model construction, data analysis, and paper writing. JM responsible for data processing and assist in writing of the paper, ZJ responsible for data processing and assist in writing of the paper. GX responsible for paper conception and method guidance.

## Funding

This paper is only funded by the National Key Research and Development Program, the sponsor is the Ministry of Science and Technology of the People’s Republic of China. The Ministry of Science and Technology of the People’s Republic of China through the key research and development plan (2018YFB1502200) funded authors to carry out the research work of the establishment of the multi-physics coupling model of the kW-level SOFC reactor. The National Key Research and Development Program funded all open access publication fees (2950USD). The study is supported by the National Key R&D Program of China 2018YFB1502200.

## Conflict of Interest

The authors declare that the research was conducted in the absence of any commercial or financial relationships that could be construed as a potential conflict of interest.

## Symbol Description

$A_{v}$: Surface area to volume ratio/m^2^⋅m^−3^
$C_{p}$: Specific heat/J⋅mol^−1^⋅K^−1^
$D_{eff,ij}$: Effective binary diffusion/m^2^⋅s^−1^
$E_{a}$: Activation energy/J⋅mol^−1^
$E_{Nernst}$: Open circuit Nernst voltage/V
F: Faraday constant/C⋅mol^−1^
M: Molar mass/kg⋅mol^−1^
R: General gas constant/J⋅mol^−1^⋅K^−1^
S: Source term
T: Temperature/K
$V_{op}$: Output voltage/V
V: Volume fraction
$i_{0}$: Exchange current density/A⋅m^−2^
k: Thermal conductivity/W⋅m^−1^⋅K^−1^
n: Number of species (in the gas-phase species transport governing equation)
u: Velocity/m⋅s^−1^
ε: Porosity
η: Polarization/V
κ: Permeability/m^2^
μ: Dynamic viscosity/kg⋅m^−1^⋅s^−1^
ρ: Density/kg⋅m^−3^
σ: Ion/Electron conductivity/S⋅m^−1^
τ: Tortuosity factor
φ: Electric potential/V
